# The genome sequence of the Scalloped Hook-tip moth, *Falcaria lacertinaria *(Linnaeus, 1758)

**DOI:** 10.12688/wellcomeopenres.23258.1

**Published:** 2024-11-07

**Authors:** Andy Griffiths, Tom Prescott, Darwin Tree of Life Barcoding collective, Wellcome Sanger Institute Tree of Life Management, Samples and Laboratory team, Wellcome Sanger Institute Scientific Operations: Sequencing Operations, Wellcome Sanger Institute Tree of Life Core Informatics team, Tree of Life Core Informatics collective, Darwin Tree of Life Consortium

**Affiliations:** 1 Wellcome Sanger Institute, Hinxton, England, UK; 2 Royal Botanic Garden Edinburgh, Edinburgh, Scotland, UK; 3 Butterfly Conservation Scotland, Stirling, Scotland, UK

**Keywords:** Falcaria lacertinaria, Scalloped Hook-tip moth, genome sequence, chromosomal, Lepidoptera

## Abstract

We present a genome assembly from an individual female *Falcaria lacertinaria* (the Scalloped Hook-tip; Arthropoda; Insecta; Lepidoptera; Drepanidae). The genome sequence has a total length of 300.20 megabases. Most of the assembly is scaffolded into 32 chromosomal pseudomolecules, including the W and Z sex chromosomes. The mitochondrial genome has also been assembled and is 16.07 kilobases in length. Gene annotation of this assembly on Ensembl identified 11,709 protein-coding genes.

## Species taxonomy

Eukaryota; Opisthokonta; Metazoa; Eumetazoa; Bilateria; Protostomia; Ecdysozoa; Panarthropoda; Arthropoda; Mandibulata; Pancrustacea; Hexapoda; Insecta; Dicondylia; Pterygota; Neoptera; Endopterygota; Amphiesmenoptera; Lepidoptera; Glossata; Neolepidoptera; Heteroneura; Ditrysia; Obtectomera; Drepanoidea; Drepanidae; Drepaninae; *Falcaria*; *Falcaria lacertinaria* (Linnaeus, 1758) (NCBI:txid505411).

## Background

**Figure 1.**
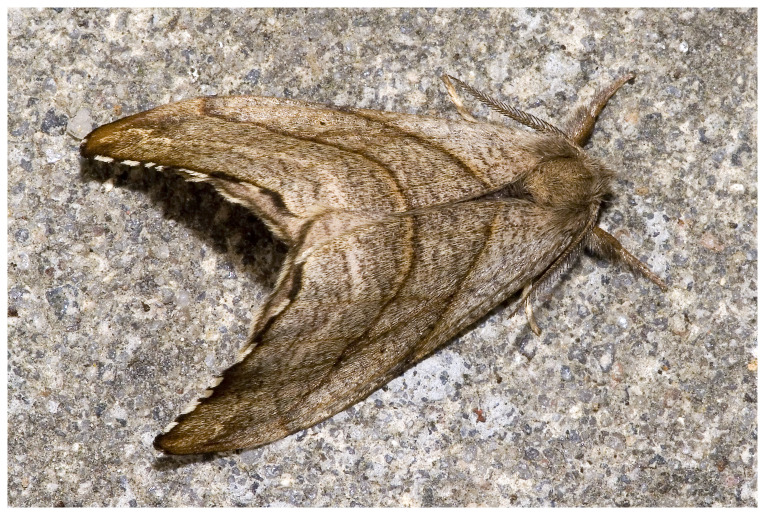
Photograph of *Falcaria lacertinaria* by Olei (not the specimen used for genome sequencing).

*Falcaria lacertinaria* (Scalloped Hook-tip) ( Figure 1) is a member of the Drepanidae family, distributed widely across Europe, including the UK and Ireland, and extending into parts of Asia ( GBIF Secretariat, 2024). It is a distinctive-looking moth, the forewing having a scalloped outer edge and chequered fringe, two dark parallel cross-lines and a central dot ( Waring *et al.*, 2017). It rests with its wings raised tent-like, looking rather like a dried leaf ( Kimber, 2024).

This species favours broadleaved woodlands, where it is associated with downy and silver birch ( *Betula spp.*), which serve as its larval foodplants. In the UK, there are two generations in summer, except in Scotland, where there is only one generation ( Waring *et al.*, 2017).

The genome of the scalloped hook-tip, *Falcaria lacertinaria*, was sequenced as part of the Darwin Tree of Life Project, a collaborative effort to sequence all named eukaryotic species in the Atlantic Archipelago of Britain and Ireland ( Blaxter *et al.*, 2022).

## Genome sequence report

The genome of an adult female specimen of *Falcaria lacertinaria* was sequenced using Pacific Biosciences single-molecule HiFi long reads, generating a total of 24.19 Gb (gigabases) from 2.09 million reads, providing approximately 78-fold coverage. Primary assembly contigs were scaffolded with chromosome conformation Hi-C data, which produced 115.16 Gbp from 762.63 million reads, yielding an approximate coverage of 384-fold. The specimen and sequencing details are summarised in Table 1.

**Table 1.** Specimen and sequencing data for *Falcaria lacertinaria*.

Project information			
**Study title**	*Falcaria lacertinaria* (scalloped hook-tip)		
**Umbrella BioProject**	PRJEB61361		
**Species**	*Falcaria lacertinaria*		
**BioSample**	SAMEA112198536		
**NCBI taxonomy ID**	505411		
Specimen information			
**Technology**	**ToLID**	**BioSample** ** accession**	**Organism part**
**PacBio long read sequencing**	ilFalLace1	SAMEA112198578	thorax
**Hi-C sequencing**	ilFalLace1	SAMEA112198578	thorax
**RNA sequencing**	ilFalLace1	SAMEA112198579	abdomen
Sequencing information			
**Platform**	**Run** **accession**	**Read count**	**Base count (Gb)**
**Hi-C Illumina NovaSeq 6000**	ERR11242564	7.63e+08	115.16
**PacBio Sequel IIe**	ERR11242140	2.09e+06	24.19
**RNA Illumina NovaSeq 6000**	ERR11837484	6.67e+07	10.08

Assembly errors were corrected by manual curation, including 23 missing joins or mis-joins. This reduced the scaffold number by 14.29%. The final assembly has a total length of 300.20 Mb in 35 sequence scaffolds with a scaffold N50 of 10.4 Mb, and 59 gaps ( Table 2).

**Table 2.** Genome assembly data for *Falcaria lacertinaria*, ilFalLace1.1.

Genome assembly		
Assembly name	ilFalLace1.1	
Assembly accession	GCA_951449985.1	
*Accession of alternate haplotype*	*GCA_951449975.1*	
Span (Mb)	300.20	
Number of contigs	95	
Contig N50 length (Mb)	4.9	
Number of scaffolds	35	
Scaffold N50 length (Mb)	10.4	
Longest scaffold (Mb)	13.98	
Assembly metrics *		*Benchmark*
Consensus quality (QV)	66.2	*≥ 50*
*k*-mer completeness	100.0%	*≥ 95%*
BUSCO **	C:98.7%[S:98.2%,D:0.5%], F:0.3%,M:1.0%,n:5,286	*C ≥ 95%*
Percentage of assembly mapped to chromosomes	99.98%	*≥ 90%*
Sex chromosomes	WZ	*localised homologous pairs*
Organelles	Mitochondrial genome: 16.07 kb	*complete single alleles*
Genome annotation of assembly GCA_951449985.1 at Ensembl		
Number of protein-coding genes	11,709	
Number of non-coding genes	1,473	
Number of gene transcripts	20,987	

* Assembly metric benchmarks are adapted from column VGP-2020 of “Table 1: Proposed standards and metrics for defining genome assembly quality” from Rhie *et al.* (2021). ** BUSCO scores based on the lepidoptera_odb10 BUSCO set using version 5.3.2. C = complete [S = single copy, D = duplicated], F = fragmented, M = missing, n = number of orthologues in comparison. A full set of BUSCO scores is available at https://blobtoolkit.genomehubs.org/view/ilFalLace1_1/dataset/ilFalLace1_1/busco.

**Figure 2.**
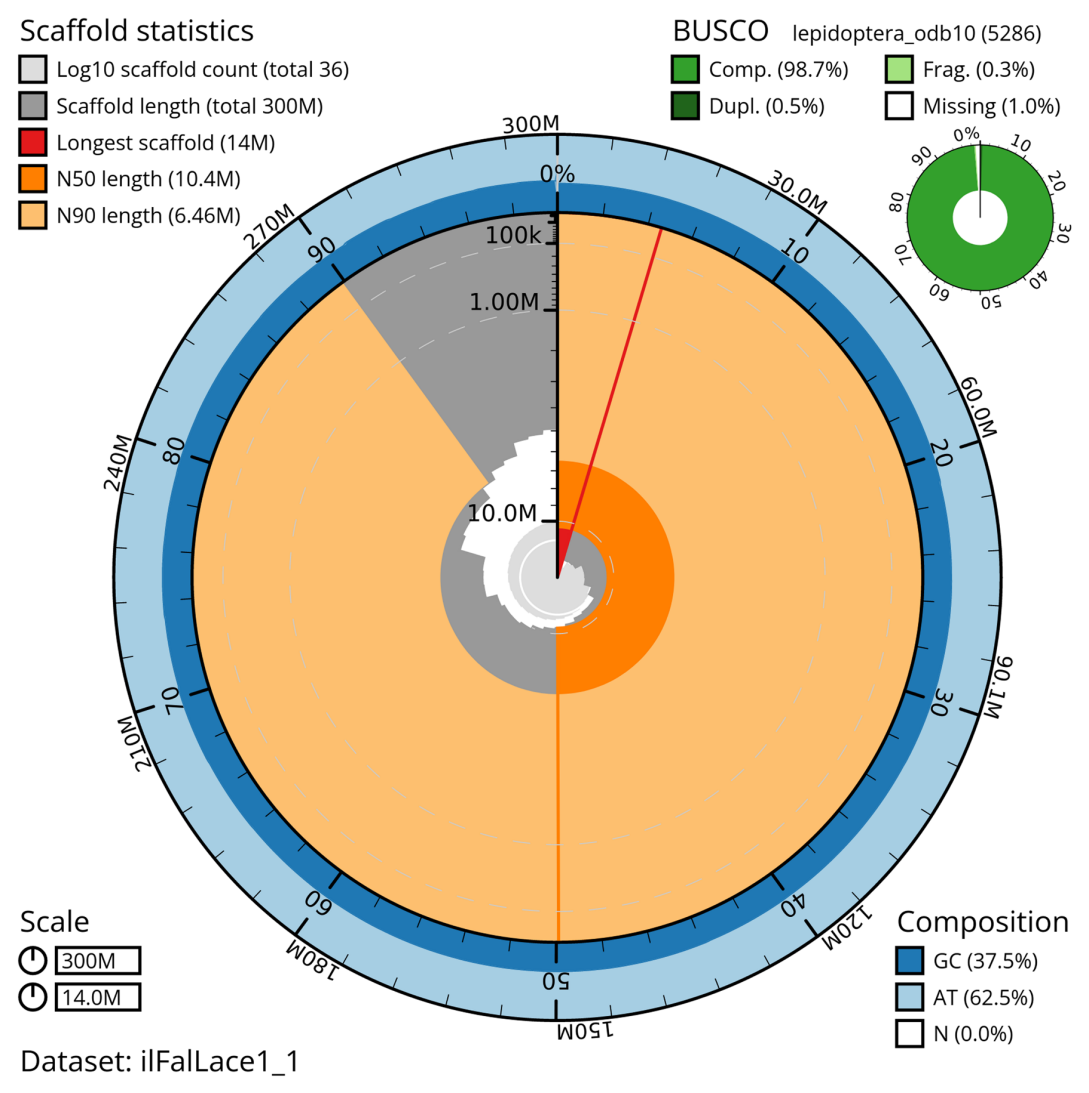
Genome assembly of *Falcaria lacertinaria*, ilFalLace1.1: metrics. The BlobToolKit snail plot shows N50 metrics and BUSCO gene completeness. The main plot is divided into 1,000 size-ordered bins around the circumference with each bin representing 0.1% of the 300,186,580 bp assembly. The distribution of scaffold lengths is shown in dark grey with the plot radius scaled to the longest scaffold present in the assembly (13,977,784 bp, shown in red). Orange and pale-orange arcs show the N50 and N90 scaffold lengths (10,359,755 and 6,461,305 bp), respectively. The pale grey spiral shows the cumulative scaffold count on a log scale with white scale lines showing successive orders of magnitude. The blue and pale-blue area around the outside of the plot shows the distribution of GC, AT and N percentages in the same bins as the inner plot. A summary of complete, fragmented, duplicated and missing BUSCO genes in the lepidoptera_odb10 set is shown in the top right. An interactive version of this figure is available at https://blobtoolkit.genomehubs.org/view/ilFalLace1_1/dataset/ilFalLace1_1/snail.

**Figure 3.**
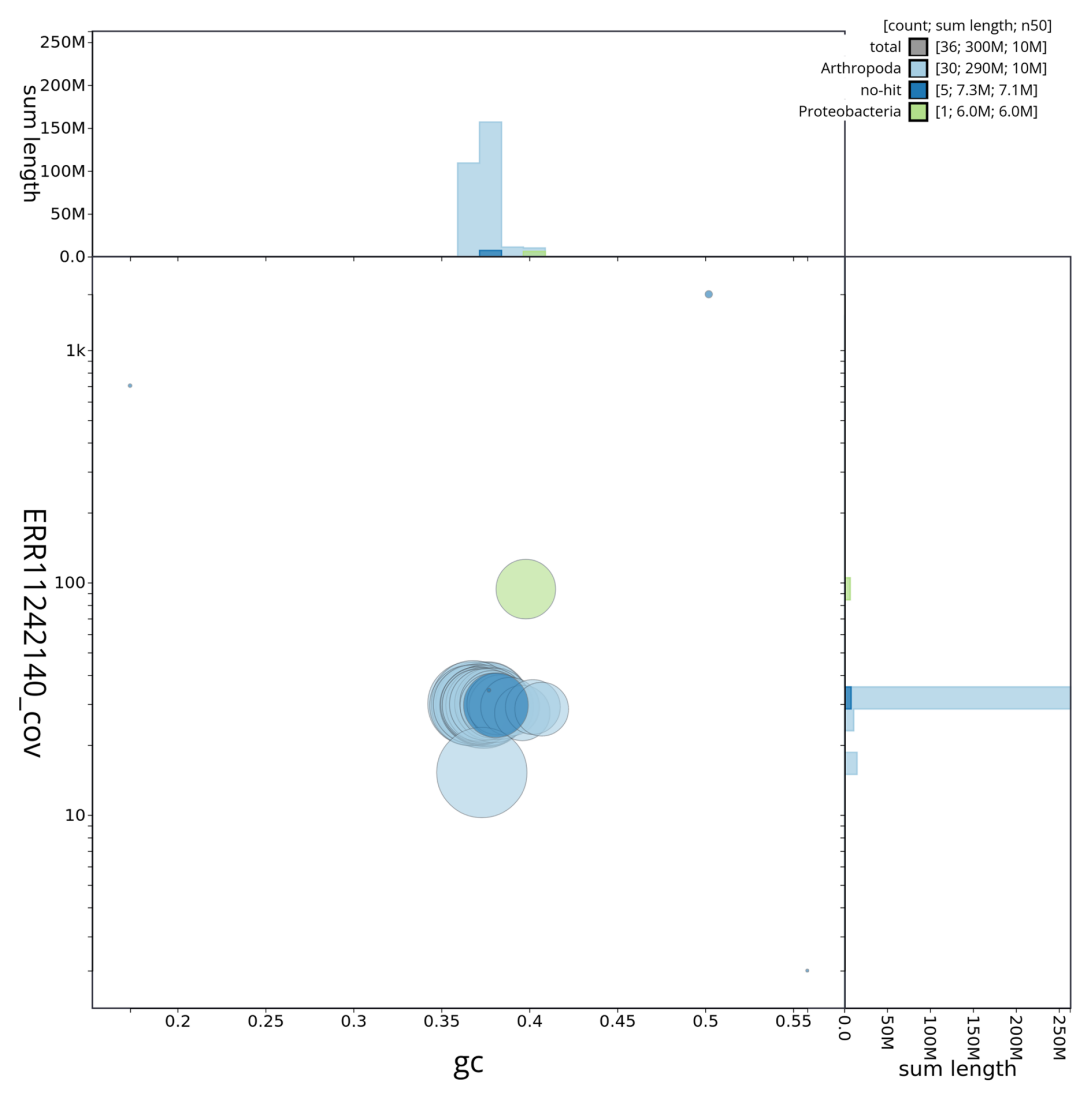
Genome assembly of *Falcaria lacertinaria*, ilFalLace1.1: BlobToolKit GC-coverage plot showing sequence coverage (vertical axis) and GC content (horizontal axis). The circles represent scaffolds, with the size proportional to scaffold length and the colour representing phylum membership. The histograms along the axes display the total length of sequences distributed across different levels of coverage and GC content. An interactive version of this figure is available at https://blobtoolkit.genomehubs.org/view/ilFalLace1_1/dataset/ilFalLace1_1/blob.

**Figure 4.**
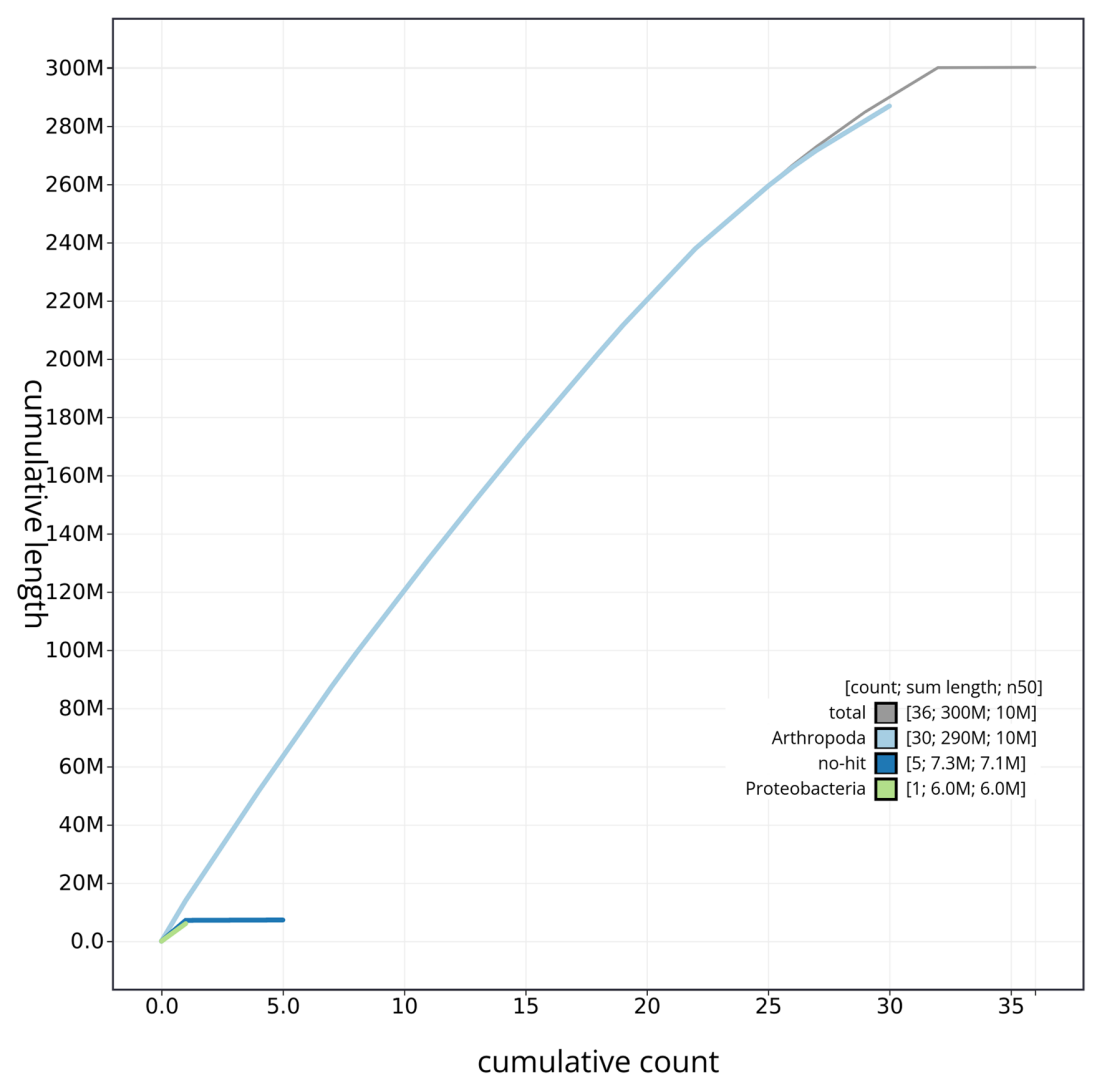
Genome assembly of *Falcaria lacertinaria* ilFalLace1.1: BlobToolKit cumulative sequence plot. The grey line shows cumulative length for all sequences. Coloured lines show cumulative lengths of sequences assigned to each phylum using the buscogenes taxrule. An interactive version of this figure is available at https://blobtoolkit.genomehubs.org/view/ilFalLace1_1/dataset/ilFalLace1_1/cumulative.

**Figure 5.**
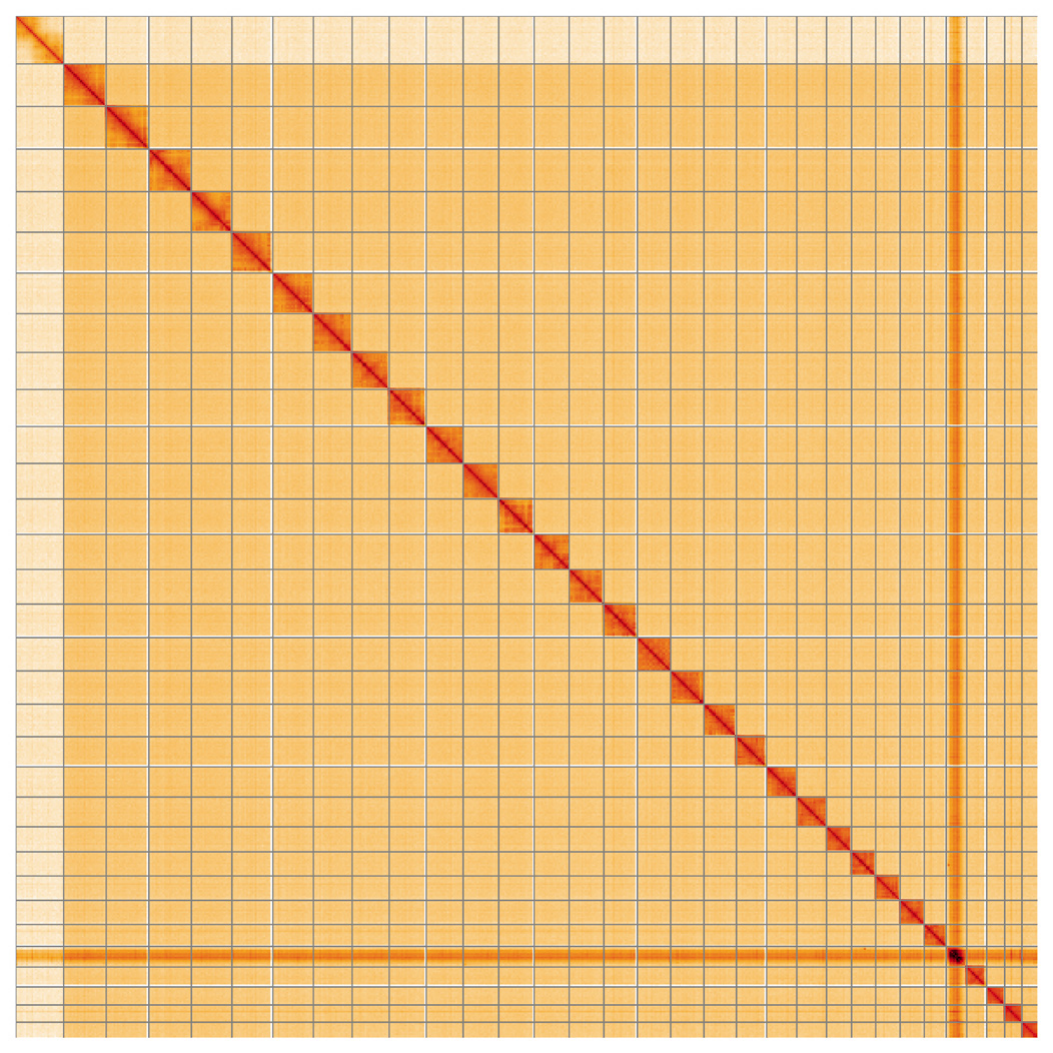
Genome assembly of *Falcaria lacertinaria* ilFalLace1.1: Hi-C contact map of the ilFalLace1.1 assembly, visualised using HiGlass. Chromosomes are shown in order of size from left to right and top to bottom. An interactive version of this figure may be viewed at https://genome-note-higlass.tol.sanger.ac.uk/l/?d=BySxyh6RRfmQPM9wDflZ2w.

The snail plot in Figure 2 provides a summary of the assembly statistics, while the distribution of assembly scaffolds on GC proportion and coverage is shown in Figure 3. The cumulative assembly plot in Figure 4 shows curves for subsets of scaffolds assigned to different phyla. Most of the assembly sequence (99.98%) was assigned to 32 chromosomal-level scaffolds, representing 30 autosomes and the W and Z sex chromosomes. Chromosome-scale scaffolds confirmed by the Hi-C data are named in order of size ( Figure 5; Table 3).

**Table 3.** Chromosomal pseudomolecules in the genome assembly of *Falcaria lacertinaria*, ilFalLace1.

INSDC accession	Name	Length (Mb)	GC%
OX602109.1	1	12.56	37.0
OX602110.1	2	12.52	37.5
OX602111.1	3	12.44	37.5
OX602112.1	4	11.96	37.5
OX602113.1	5	11.93	36.5
OX602114.1	6	11.9	37.0
OX602115.1	7	11.37	37.0
OX602116.1	8	10.89	36.5
OX602117.1	9	10.88	36.5
OX602118.1	10	10.78	37.0
OX602119.1	11	10.54	37.5
OX602120.1	12	10.36	37.5
OX602121.1	13	10.28	37.0
OX602122.1	14	10.12	37.0
OX602123.1	15	9.88	37.0
OX602124.1	16	9.75	37.0
OX602125.1	17	9.74	37.5
OX602126.1	18	9.54	38.0
OX602127.1	19	8.86	37.0
OX602128.1	20	8.83	38.0
OX602129.1	21	8.78	37.5
OX602130.1	22	7.29	38.0
OX602131.1	23	7.14	38.0
OX602132.1	24	7.12	38.0
OX602133.1	25	7.09	38.5
OX602134.1	26	6.46	38.0
OX602136.1	27	5.85	39.0
OX602137.1	28	5.21	39.5
OX602138.1	29	5.1	40.0
OX602139.1	30	4.9	40.5
OX602135.1	W	6.03	40.0
OX602108.1	Z	13.98	37.5
OX602140.1	MT	0.02	18.0

While not fully phased, the assembly deposited is of one haplotype. Contigs corresponding to the second haplotype have also been deposited. The mitochondrial genome was also assembled and can be found as a contig within the multifasta file of the genome submission.

The final assembly has a Quality Value (QV) of 66.2 and *k*-mer completeness of 100.0%. BUSCO (v5.3.2 ) analysis using the lepidoptera_odb10 reference set ( *n* = 5,286) indicated a completeness score of 98.7% (single = 98.2%, duplicated = 0.5%).

Metadata for specimens, BOLD barcode results, spectra estimates, sequencing runs, contaminants and pre-curation assembly statistics are given at https://links.tol.sanger.ac.uk/species/505411.

### Genome annotation report

The *Falcaria lacertinaria* genome assembly (GCA_951449985.1) was annotated at the European Bioinformatics Institute (EBI) on Ensembl Rapid Release. The resulting annotation includes 20,987 transcribed mRNAs from 11,709 protein-coding and 1,473 non-coding genes ( Table 2; https://rapid.ensembl.org/Falcaria_lacertinaria_GCA_951449985.1/Info/Index). The average transcript length is 12,893.38. There are 1.59 coding transcripts per gene and 7.91 exons per transcript.

## Methods

### Sample acquisition

An adult specimen of *Falcaria lacertinaria* (specimen ID SAN00002615, ToLID ilFalLace1) was collected from Glen Strathfarrar, Scotland, UK (latitude 57.41, longitude –4.73) on 2022-06-27, using a moth trap. The specimen was collected by Andy Griffiths (Wellcome Sanger Institute) and identified by Tom Prescott (Butterfly Conservation) and preserved by flash freezing.

### Nucleic acid extraction

The workflow for high molecular weight (HMW) DNA extraction at the Wellcome Sanger Institute (WSI) Tree of Life Core Laboratory includes a sequence of core procedures: sample preparation and homogenisation, DNA extraction, fragmentation and purification. Detailed protocols are available on protocols.io ( Denton *et al.*, 2023b). The ilFalLace1 sample was prepared for DNA extraction by weighing and dissecting it on dry ice ( Jay *et al.*, 2023) and thorax tissue was homogenised using a PowerMasher II tissue disruptor ( Denton *et al.*, 2023a).

HMW DNA was extracted in the WSI Scientific Operations core using the Automated MagAttract v2 protocol ( Oatley *et al.*, 2023). The DNA was sheared into an average fragment size of 12–20 kb in a Megaruptor 3 system ( Bates *et al.*, 2023). Sheared DNA was purified by solid-phase reversible immobilisation, using AMPure PB beads to eliminate shorter fragments and concentrate the DNA ( Strickland *et al.*, 2023). The concentration of the sheared and purified DNA was assessed using a Nanodrop spectrophotometer and Qubit Fluorometer using the Qubit dsDNA High Sensitivity Assay kit. Fragment size distribution was evaluated by running the sample on the FemtoPulse system.

RNA was extracted from abdomen tissue of ilFalLace1 in the Tree of Life Laboratory at the WSI using the RNA Extraction: Automated MagMax™ *mir*Vana protocol ( do Amaral *et al.*, 2023). The RNA concentration was assessed using a Nanodrop spectrophotometer and a Qubit Fluorometer using the Qubit RNA Broad-Range Assay kit. Analysis of the integrity of the RNA was done using the Agilent RNA 6000 Pico Kit and Eukaryotic Total RNA assay.

### Hi-C preparation

Tissue from the thorax of the ilFalLace1 sample was processed at the WSI Scientific Operations core, using the Arima-HiC v2 kit. Tissue (stored at –80 °C) was fixed, and the DNA crosslinked using a TC buffer with 22% formaldehyde. After crosslinking, the tissue was homogenised using the Diagnocine Power Masher-II and BioMasher-II tubes and pestles. Following the kit manufacturer's instructions, crosslinked DNA was digested using a restriction enzyme master mix. The 5’-overhangs were then filled in and labelled with biotinylated nucleotides and proximally ligated. An overnight incubation was carried out for enzymes to digest remaining proteins and for crosslinks to reverse. A clean up was performed with SPRIselect beads prior to library preparation.

### Library preparation and sequencing

Library preparation and sequencing were performed at the WSI Scientific Operations core. Pacific Biosciences HiFi circular consensus DNA sequencing libraries were prepared using the PacBio Express Template Preparation Kit v2.0 (Pacific Biosciences, California, USA) as per the manufacturer's instructions. The kit includes the reagents required for removal of single-strand overhangs, DNA damage repair, end repair/A-tailing, adapter ligation, and nuclease treatment. Library preparation also included a library purification step using AMPure PB beads (Pacific Biosciences, California, USA) and size selection step to remove templates shorter than 3 kb using AMPure PB modified SPRI. DNA concentration was quantified using the Qubit Fluorometer v2.0 and Qubit HS Assay Kit and the final library fragment size analysis was carried out using the Agilent Femto Pulse Automated Pulsed Field CE Instrument and gDNA 165kb gDNA and 55kb BAC analysis kit. Samples were sequenced using the Sequel IIe system (Pacific Biosciences, California, USA). The concentration of the library loaded onto the Sequel IIe was between 40–135 pM. The SMRT link software, a PacBio web-based end-to-end workflow manager, was used to set-up and monitor the run, as well as perform primary and secondary analysis of the data upon completion.

For Hi-C library preparation, DNA was fragmented to a size of 400 to 600 bp using a Covaris E220 sonicator. The DNA was then enriched, barcoded, and amplified using the NEBNext Ultra II DNA Library Prep Kit following manufacturers’ instructions. The Hi-C sequencing was performed using paired-end sequencing with a read length of 150 bp on an Illumina NovaSeq 6000 instrument.

Poly(A) RNA-Seq libraries were constructed using the NEB Ultra II RNA Library Prep kit, following the manufacturer’s instructions. RNA sequencing was performed on the Illumina NovaSeq 6000 instrument.

### Genome assembly, curation and evaluation

***Assembly***

The original assembly of HiFi reads was performed using Hifiasm ( Cheng *et al.*, 2021) with the --primary option. Haplotypic duplications were identified and removed with purge_dups ( Guan *et al.*, 2020). Hi-C reads are further mapped with bwamem2 ( Vasimuddin *et al.*, 2019) to the primary contigs, which are further scaffolded using the provided Hi-C data ( Rao *et al.*, 2014) in YaHS ( Zhou *et al.*, 2023) using the --break option. Scaffolded assemblies are evaluated using Gfastats ( Formenti *et al.*, 2022), BUSCO ( Manni *et al.*, 2021) and MERQURY.FK ( Rhie *et al.*, 2020).

The mitochondrial genome was assembled using MitoHiFi ( Uliano-Silva *et al.*, 2023), which runs MitoFinder ( Allio *et al.*, 2020) and uses these annotations to select the final mitochondrial contig and to ensure the general quality of the sequence.

***Assembly curation***

The assembly was decontaminated using the Assembly Screen for Cobionts and Contaminants (ASCC) pipeline (article in preparation). Flat files and maps used in curation were generated in TreeVal ( Pointon *et al.*, 2023). Manual curation was primarily conducted using PretextView ( Harry, 2022), with additional insights provided by JBrowse2 ( Diesh *et al.*, 2023) and HiGlass ( Kerpedjiev *et al.*, 2018). Scaffolds were visually inspected and corrected as described by Howe *et al*. (2021). Any identified contamination, missed joins, and mis-joins were corrected, and duplicate sequences were tagged and removed. The sex chromosomes were identified by read coverage statistics. The entire process is documented at https://gitlab.com/wtsi-grit/rapid-curation (article in preparation).

***Evaluation of the final assembly***

A Hi-C map for the final assembly was produced using bwa-mem2 ( Vasimuddin *et al.*, 2019) in the Cooler file format ( Abdennur & Mirny, 2020). To assess the assembly metrics, the *k*-mer completeness and QV consensus quality values were calculated in Merqury ( Rhie *et al.*, 2020). This work was done using the “sanger-tol/readmapping” ( Surana *et al.*, 2023a) and “sanger-tol/genomenote” ( Surana *et al.*, 2023b) pipelines. The genome evaluation pipelines were developed using nf-core tooling ( Ewels *et al.*, 2020) and MultiQC ( Ewels *et al.*, 2016), relying on the Conda package manager, the Bioconda initiative ( Grüning *et al.*, 2018), the Biocontainers infrastructure ( da Veiga Leprevost *et al.*, 2017), as well as the Docker ( Merkel, 2014) and Singularity ( Kurtzer *et al.*, 2017) containerisation solutions. The genome was also analysed within the BlobToolKit environment ( Challis *et al.*, 2020) and BUSCO scores ( Manni *et al.*, 2021) were calculated.

Table 4 contains a list of relevant software tool versions and sources.

**Table 4.** Software tools: versions and sources.

Software tool	Version	Source
BlobToolKit	4.2.1	https://github.com/blobtoolkit/blobtoolkit
BUSCO	5.3.2	https://gitlab.com/ezlab/busco
bwa-mem2	2.2.1	https://github.com/bwa-mem2/bwa-mem2
Cooler	0.8.11	https://github.com/open2c/cooler
Gfastats	1.3.6	https://github.com/vgl-hub/gfastats
Hifiasm	0.16.1-r375	https://github.com/chhylp123/hifiasm
HiGlass	1.11.6	https://github.com/higlass/higlass
Merqury	MerquryFK	https://github.com/thegenemyers/MERQURY.FK
MitoHiFi	2	https://github.com/marcelauliano/MitoHiFi
Nextflow	23.04.0-5857	https://github.com/nextflow-io/nextflow
PretextView	0.2	https://github.com/wtsi-hpag/PretextView
purge_dups	1.2.3	https://github.com/dfguan/purge_dups
sanger-tol/genomenote	v1.0	https://github.com/sanger-tol/genomenote
sanger-tol/readmapping	1.1.0	https://github.com/sanger-tol/readmapping/tree/1.1.0
YaHS	yahs-1.1.91eebc2	https://github.com/c-zhou/yahs

### Genome annotation

The Ensembl Genebuild annotation system ( Aken *et al.*, 2016) was used to generate annotation for the *Falcaria lacertinaria* assembly (GCA_951449985.1) in Ensembl Rapid Release at the EBI. Annotation was created primarily through alignment of transcriptomic data to the genome, with gap filling via protein-to-genome alignments of a select set of proteins from UniProt ( UniProt Consortium, 2019).

### Wellcome Sanger Institute – Legal and Governance

The materials that have contributed to this genome note have been supplied by a Darwin Tree of Life Partner. The submission of materials by a Darwin Tree of Life Partner is subject to the **‘Darwin Tree of Life Project Sampling Code of Practice’**, which can be found in full on the Darwin Tree of Life website here. By agreeing with and signing up to the Sampling Code of Practice, the Darwin Tree of Life Partner agrees they will meet the legal and ethical requirements and standards set out within this document in respect of all samples acquired for, and supplied to, the Darwin Tree of Life Project.

Further, the Wellcome Sanger Institute employs a process whereby due diligence is carried out proportionate to the nature of the materials themselves, and the circumstances under which they have been/are to be collected and provided for use. The purpose of this is to address and mitigate any potential legal and/or ethical implications of receipt and use of the materials as part of the research project, and to ensure that in doing so we align with best practice wherever possible. The overarching areas of consideration are:

•   Ethical review of provenance and sourcing of the material

•   Legality of collection, transfer and use (national and international)

Each transfer of samples is further undertaken according to a Research Collaboration Agreement or Material Transfer Agreement entered into by the Darwin Tree of Life Partner, Genome Research Limited (operating as the Wellcome Sanger Institute), and in some circumstances other Darwin Tree of Life collaborators.

## Data Availability

European Nucleotide Archive: *Falcaria lacertinaria* (scalloped hook-tip). Accession number PRJEB61361; https://identifiers.org/ena.embl/PRJEB61361 ( Wellcome Sanger Institute, 2023). The genome sequence is released openly for reuse. The *Falcaria lacertinaria* genome sequencing initiative is part of the Darwin Tree of Life (DToL) project. All raw sequence data and the assembly have been deposited in INSDC databases. Raw data and assembly accession identifiers are reported in Table 1 and Table 2.

## Author information

Members of the Darwin Tree of Life Barcoding collective are listed here: https://doi.org/10.5281/zenodo.12158331

Members of the Wellcome Sanger Institute Tree of Life Management, Samples and Laboratory team are listed here: https://doi.org/10.5281/zenodo.12162482.

Members of Wellcome Sanger Institute Scientific Operations: Sequencing Operations are listed here: https://doi.org/10.5281/zenodo.12165051.

Members of the Wellcome Sanger Institute Tree of Life Core Informatics team are listed here: https://doi.org/10.5281/zenodo.12160324.

Members of the Tree of Life Core Informatics collective are listed here: https://doi.org/10.5281/zenodo.12205391.

Members of the Darwin Tree of Life Consortium are listed here: https://doi.org/10.5281/zenodo.4783558.
