# Supplementary material for: The genome sequence of the Scalloped Hook-tip moth, Falcaria lacertinaria (Linnaeus, 1758)
Source: Wellcome Open Res. 2024 Nov 7;9:659. [Version 1] doi: 10.12688/wellcomeopenres.23258.1 (PMC11624438; doi:10.12688/wellcomeopenres.23258.1)
Supplement: Peer review history

# Reviewer report: Reviewer response for version 1

**DOI:** 10.21956/wellcomeopenres.25636.r110543

**Published:** 2024-12-07

**Authors:** Sina Beier

**Affiliations:** 1 Gurdon Institute (Ringgold ID: 47693), Cambridge, England, UK

The presented article provides a high quality curated assembly and annotation of the scalloped hook-tip moth genome. While the methodology is well described, it would be helpful for future publications to provide supplementary information on the manual curation of the assembly including a mapping of the curated loci in the final genome to improve the reproducibility of the approach and usability of the genome for future research.

Are sufficient details of methods and materials provided to allow replication by others?

Partly

Is the rationale for creating the dataset(s) clearly described?

Yes

Are the datasets clearly presented in a useable and accessible format?

Yes

Are the protocols appropriate and is the work technically sound?

Yes

Reviewer Expertise:

bioinformatics, assembly of large genomes, genome annotation

I confirm that I have read this submission and believe that I have an appropriate level of expertise to confirm that it is of an acceptable scientific standard.

# Reviewer report: Reviewer response for version 1

**DOI:** 10.21956/wellcomeopenres.25636.r110538

**Published:** 2024-12-06

**Authors:** Marko Mutanen

**Affiliations:** 1 University of Oulu, Oulu, Finland

This article provides a genome sequence for a moth *Falcaria lacertinaria*, a member of the lepidopteran family Drepanidae and its subfamily Drepaninae, a group which is not species rich in Europe. The species is unmistakable in the area suggesting that the identification of the specimen must be correct although no images of the voucher are provided. The sequenced specimen is a female which is good as both W and Z chromosomes are included. Also full mitochondrial genome sequence is provided.

 To my best understanding, the genome quality is very high. The analyzed specimen was snap-frozen that ensured the high quality of specimen DNA. The methods used follow the established yet continuously developing standards of the Wellcome Sanger Institute. The snail plot figure as well as other graphs, figures and plots demonstrate the high quality of the genome. For example, 98.7% of the BUSCO loci of the Lepidoptera reference set of with 5286 loci were recovered which is a very high number.

Are sufficient details of methods and materials provided to allow replication by others?

Yes

Is the rationale for creating the dataset(s) clearly described?

Yes

Are the datasets clearly presented in a useable and accessible format?

Yes

Are the protocols appropriate and is the work technically sound?

Yes

Reviewer Expertise:

Lepidoptera and sawfly molecular taxonomy, systematics and phylogenetics. Insects biodiversity.

I confirm that I have read this submission and believe that I have an appropriate level of expertise to confirm that it is of an acceptable scientific standard.

# Reviewer report: Reviewer response for version 1

**DOI:** 10.21956/wellcomeopenres.25636.r110544

**Published:** 2024-11-21

**Authors:** Shiqi Luo

**Affiliations:** 1 Department of Entomology, College of Plant Protection, China Agricultural University, Beijing, China

This article describes the chromosomal-level genome data of the Scalloped Hook-tip moth *Falcaria lacertinaria*, obtained through a combination of PacBio long read sequencing, Hi-C sequencing, and RNA-seq. The bioinformatics analysis techniques used are sound, leading to a high assembly quality. The article also includes comprehensive information and experimental analysis details, presented in a clear manner.

 I have 3 questions

 1) Is the gut removed when sequencing RNA in the abdomen, given that it contains a significant amount of gut bacteria? Could the presence of bacterial transcripts in the whole abdomen transcriptome affect the subsequent analysis?

 2) Where is the bacterial sequence located in the genome according to the analysis results obtained using BlobTool, and should it be removed considering that 6.0 Mb of sequences belong to Proteobacteria?

 3) Is it possible to add annotations of transposable elements to the existing annotations of protein-coding genes and non-coding genes in the article?

Are sufficient details of methods and materials provided to allow replication by others?

Yes

Is the rationale for creating the dataset(s) clearly described?

Yes

Are the datasets clearly presented in a useable and accessible format?

Yes

Are the protocols appropriate and is the work technically sound?

Yes

Reviewer Expertise:

functional genomics, gut microbiome

I confirm that I have read this submission and believe that I have an appropriate level of expertise to confirm that it is of an acceptable scientific standard.
